# The use of concentrated growth factors in guiding bone regeneration after microsurgical endodontic surgery for periapical lesions

**DOI:** 10.1093/rb/rbaf058

**Published:** 2025-06-23

**Authors:** Qi Lin, Shaofeng Liu, Minmin Wang, Zhongxiong Ma, Bin Shi

**Affiliations:** Department of Endodontics, Fujian Key Laboratory of Oral Diseases & Fujian Provincial Engineering Research Center of Oral Biomaterial & Stomatological Key Laboratory of Fujian College and University, School and Hospital of Stomatology, Fujian Medical University, 246 Yangqiao Zhong Road, Fuzhou, Fujian, 350001, People's Republic of China; Department of Oral and Maxillofacial Surgery, The First Affiliated Hospital of Fujian Medical University, 20 Cha Zhong Road, Fuzhou, Fujian, 350002, People's Republic of China; Department of Endodontics, Fujian Key Laboratory of Oral Diseases & Fujian Provincial Engineering Research Center of Oral Biomaterial & Stomatological Key Laboratory of Fujian College and University, School and Hospital of Stomatology, Fujian Medical University, 246 Yangqiao Zhong Road, Fuzhou, Fujian, 350001, People's Republic of China; Department of Endodontics, Fujian Key Laboratory of Oral Diseases & Fujian Provincial Engineering Research Center of Oral Biomaterial & Stomatological Key Laboratory of Fujian College and University, School and Hospital of Stomatology, Fujian Medical University, 246 Yangqiao Zhong Road, Fuzhou, Fujian, 350001, People's Republic of China; Department of Oral and Maxillofacial Surgery, The First Affiliated Hospital of Fujian Medical University, 20 Cha Zhong Road, Fuzhou, Fujian, 350002, People's Republic of China

**Keywords:** concentrated growth factors, biomaterial, scaffold, guided bone regeneration

## Abstract

Concentrated growth factors (CGFs) hold great potentials for postoperative bone regeneration. This study attempted to investigate the effect of CGF scaffolds on guided bone regeneration after microsurgical endodontic surgery on teeth with periapical lesions. Microsurgical endodontic surgery was performed on 68 teeth with periapical lesions after complete root canal therapy. Autologous CGFs were administered to 38 teeth (the experimental group) while the remaining teeth received no CGF (the control group). The patients were followed for an average of 18 months. Postoperative pain, swelling and the duration were compared between the two groups. The bone volume ratios were quantitatively measured and statistically analyzed with Mimics software. Compared with the control group, the experimental group reported a lower incidence and shorter duration of postoperative pain and swelling, with mild to moderate swelling in the former and mild swelling in the latter. Both groups demonstrated good postoperative wound healing. The experimental group reported a significant reduction in bone volume ratio at postoperative month 3 (*P* < 0.05). Both groups reported a most active period of new bone formation between 3 and 6 postoperative months, after which the formation rate stabilized, and an insignificant decrease in bone volume ratio from 6 to 18 postoperative months. By 18 postoperative months, the bone defects were minimized, with the experimental group showing faster new bone formation. Marked differences in bone volume reduction and volume reduction rate were found between the two groups, with more significant bone defect repair and bone regeneration in the experimental group. These results evidence that in guided bone regeneration, the use of CGF scaffolds for teeth with periapical lesions can alleviate postoperative pain and swelling, promote faster bone defect repair and ensure satisfactory incision healing, highlighting it as a promising clinical approach.

## Graphical Abstract

**Figure:**
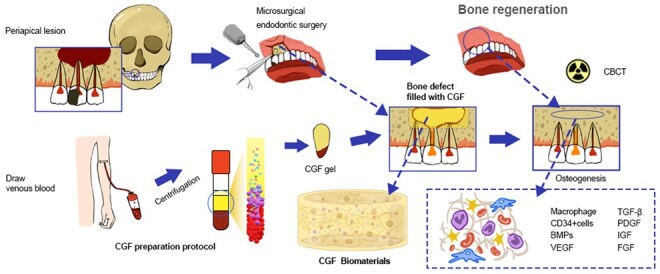

## Introduction

Periapical periodontitis is a common chronic inflammation that results from the spread of pulp infection to the periapical tissues. It is characterized by inflammatory responses and localized bone destruction around the root apex. During disease progression, persistent infection may occur due to the host immune response to the pathogenic stimuli within the root canal. If the infection is not thoroughly eradicated, pathogens will continue to invade the periapical tissues, resulting in a chronic inflammation, treatment complexity and prolonged healing process.

Clinically, acute periapical periodontitis primarily features swelling and pain in the affected tooth and surrounding tissues, whereas chronic periapical periodontitis is mainly characterized by alveolar bone loss around the root apex and the formation of inflammatory granulation tissue. The latter, rich in lymphocytes and fibroblasts, helps limit the spread of infection and maintain the local defense. However, when this defense is disrupted, the decrease of fibrous components and increase of inflammatory cells and capillary permeability promote osteoclastic activity, leading to progressive bone destruction. As the disease advances, the extent of bone loss will gradually increase and ultimately impact the prognosis of the affected teeth.

In most cases, conventional root canal therapy can eliminate pathogenic irritants and promote lesion healing. However, a subset of resistant microorganisms, such as *Enterococcus faecalis*, *Propionibacterium* and *Actinomyces* spp., may persist in the periapical area [1], thus, preventing a complete resolution. Studies report that periapical lesions mainly consist of granulomas (77%), cysts (18%), abscesses (3%) and scar tissue (2%) [2]. Except for the scar tissue, most lesions require surgical intervention to remove the infected tissue. However, while traditional apical surgery can eliminate the lesions, it remains a significant challenge to remedy large bone defects.

Currently, the treatment of periapical lesions is routinely evaluated by radiographic means. For instance, cone-beam computed tomography (CBCT) can effectively display a three-dimensional bone destruction and the relation between lesions and adjacent anatomical structures, providing essential information for preoperative risk assessment and surgical planning [3]. Additionally, digital reconstruction software combined with CBCT imaging can precisely assess the postoperative new bone deposition, facilitating a quantitative assessment of treatment efficacy.

Meanwhile, to address the large bone defect regeneration, the guided bone regeneration (GBR) technology has been widely employed. By combining biomaterials and growth factors, GBR significantly facilitates bone repair and regeneration, offering a promising strategy for managing bone defects. Of all the GBR approaches, platelet-derived products [4] hold a great promise due to their high content of growth factors that are critical for wound healing and tissue repair [5, 6], including vascular endothelial growth factor (VEGF) and transforming growth factor-beta (TGF-β).

The first-generation platelet product, platelet-rich plasma (PRP), has been demonstrated to promote soft tissue healing and facilitate periodontal regeneration surgeries [7, 8]. However, its application to hard tissue regeneration is largely compromised by factors such as the requirement of exogenous thrombin during preparation, the short release period of growth factors and significant individual variability. As a second-generation platelet product [9, 10], platelet-rich fibrin (PRF), is produced through centrifugation without anticoagulants, forming a three-dimensional fibrin network capable of sustained growth factor release, thereby promoting soft tissue repair [11]. Nevertheless, the efficacy of PRF is limited in addressing large-volume bone defects.

Introduced in 2006, concentrated growth factor (CGF), a third-generation platelet product obtained via variable-speed centrifugation, is rich in growth factors and possesses a dense, elastic fibrin matrix, providing an optimal microenvironment for cell attachment and proliferation while enhancing angiogenesis and osteogenic differentiation [12]. Importantly, without the involvement of any exogenous agents, CGF boasts excellent biocompatibility and can be applied alone or in combination with various biomaterials for bone tissue engineering [12, 13]. Compared with PRP and PRF, CGF exhibits superior composition stability, mechanical properties and regenerative potential [14].

Since introduction, CGF has been successfully applied to peri-implant bone regeneration, alveolar bone augmentation and periodontal tissue regeneration [15, 16]. In the fields of pulp and periodontal regeneration, CGF has been documented to promote angiogenesis and fibroblast proliferation while maintaining favorable biocompatibility and low immunogenicity [17, 18]. Preliminary studies have also explored the role of CGF in repairing periapical tissue defects [19, 20]. However, the generalizability of these findings is compromised due to limitations such as small sample sizes and a lack of comprehensive, quantitative clinical evaluations.

Given these considerations, the present study aimed to provide a systematic assessment of the clinical effects of CGF on periapical tissue regeneration after microsurgical endodontic surgery by CBCT-based quantitative analysis. Specifically, postoperative changes in bone defect volume, pain and swelling were comprehensively evaluated to elucidate the potential of CGF in promoting periapical tissue regeneration. The findings may provide novel insights into the clinical value of CGF in treating periapical large bone defects.

## Materials and methods

### Clinical samples

A total of 56 patients, who visited the Department of Endodontics, Affiliated Stomatological Hospital of Fujian Medical University, between October 2021 and October 2023, were diagnosed with periapical lesions involving 68 affected teeth. After root canal therapy, all patients underwent microsurgical endodontic surgery. This study was approved by the Ethics Committee of the Affiliated Stomatological Hospital of Fujian Medical University (Approval No.: 2020).

### Clinical grouping

All patient records were complete and a total of 68 teeth treated with microsurgical endodontic surgery were included. Of them, 38 teeth (15 from males and 23 from females, with an age range of 13–57 years) received microsurgical endodontic surgery and subsequent guided bone regeneration (GBR) with autologous blood-derived CGFs (the Experimental group), in which 30 lesions were located in the maxilla and 8 in the mandible; the remaining 30 teeth (5 from males and 25 from females, with an age range of 13–52 years) only underwent microsurgical endodontic surgery, without the use of CGFs (the Control group), in which 22 lesions were located in the maxilla and 8 in the mandible. All patients provided written informed consent prior to participation in the study.

As this study was a non-randomized control trial, to minimize bias due to insufficient sample size, a sample size calculation was performed by a two-sided α of 0.05 and a power of 90%:


$$n=\frac{2{\left(z_{\alpha }+z_{\beta }\right)}^{2}*\sigma^{2}}{\delta^{2}}.$$

The calculation yielded a required sample size of 24 cases per group, assuming a 1:1 ratio between the intervention and control groups. Considering a potential 20% loss to follow-up or refusal to participate, the final required number of subjects was adjusted to 30 per group, producing a total of at least 60 participants. With the included 68 cases, the study protocol met the required sample size standard.

### Inclusion and exclusion criteria

#### Inclusion criteria

- Patients with periapical lesions who still presented symptoms or signs after thorough root canal treatment or retreatment, with CBCT showing well-defined radiolucent areas in the jawbone, and a minimum lesion diameter of ≥10 mm; lesions with complete or partial buccal/lingual or palatal bone loss; or lesions involving adjacent teeth.
- Patients with no systemic diseases, good surgical anesthetic tolerance and good compliance.
- Patients with good cooperativeness with the surgical operator.
- Patients without serious cardiovascular, pulmonary or neurological diseases.
- Patients with normal liver and kidney function.
- Patients with normal coagulation function.
- Patients without oral mucosal diseases.

#### Exclusion criteria

- Patients with systemic diseases that affect bone healing and regeneration.
- Patients with acute infections.
- Patients with severe local or systemic diseases, such as malignancies, moderate to severe cardiovascular diseases, liver or kidney diseases, uncontrolled diabetes, untreated hematologic disorders or coagulopathies or severe asthma.
- Pregnant women, patients with mental disorders or those unable to undergo radiographic examination or maintain oral hygiene.
- Patients with a history of bisphosphonate use or receiving radiotherapy or chemotherapy within the past six months.
- Patients reporting incomplete clinical data, poor compliance, inability to attend follow-up appointments or unwillingness to participate in the study.

### Clinical treatment process

#### Preoperative preparation

Patients diagnosed with periapical lesions at their initial visit were examined by preoperative CBCT (i-CAT 17-19, KaVo, USA). The scanning parameters were set as follows: tube voltage 120 kV, tube current 5 mA, exposure time 7 s/rotation, scan diameter 16 cm, scan height 10 cm, slice thickness 0.2 mm, voxel size 0.2 × 0.2 mm. The 3D voxel size was *X*-0.2 mm, *Y*-0.2 mm and *Z*-0.2 mm. The lesion location, boundaries and extent were measured. For cases involving the pulp or periapical lesion, complete root canal therapy was performed; for cases of incomplete root canal treatment, retreatment was conducted. Routine full-mouth scaling was also performed before surgery. All root canal treatments were independently completed by a clinician with 10 years of experience.

#### Preoperative discussion

The patient was informed of the potential risks associated with the surgery, including the need to extract teeth with lesions deemed non-salvageable during the procedure. Postoperative complications were also explained in detail. The patient and their family granted their informed consent before the surgery.

#### CGF preparation

Ten minutes before surgery, 9 ml of venous blood was drawn from patients in the experimental group into a vacuum blood collection tube. The tube was placed in a centrifuge (Medifuge 200, Silfradent, Italy). The CGF preparation protocol was carried out as follows: the centrifuge accelerated for 30 s to reach a speed of 2700 rpm for 2 min, subsequently decelerated to 2400 rpm for 4 min, and then, accelerated again to 2700 rpm for 4 min and to 3300 rpm for 3 min. The process concluded with a 36-s deceleration to a stop. After centrifugation, the blood sample was separated into three layers and was stored at 4°C before use.

#### Surgical procedure

The surgery in all cases was performed by two experienced endodontic microsurgeons in a designated operating room equipped with a surgical microscope (OMS2380, Zumax, China). The surgical protocol followed the guidelines of the American Association of Endodontists (AAE) [21].

**Figure 1.**
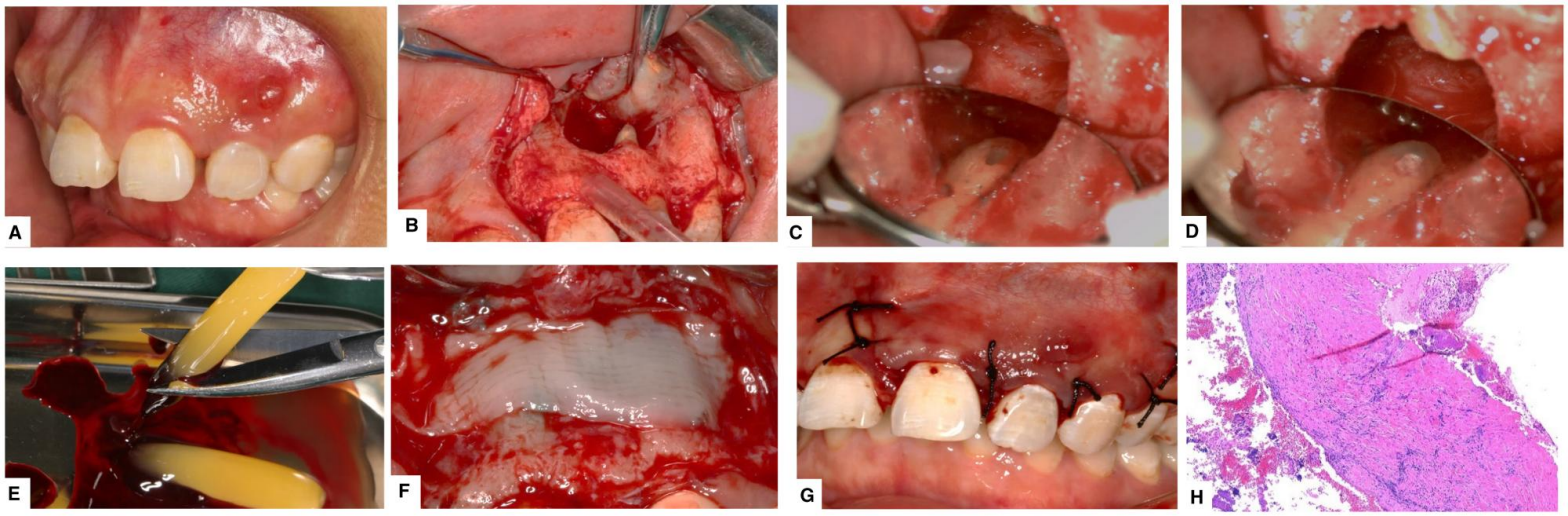
Steps of microscopic endodontic surgery. (**A**) Preoperative intraoral photograph. (**B**) Incision and reflection of the full-thickness mucoperiosteal flap, followed by detachment of the cystic wall. (**C**) Microscopically guided resection of 3 mm of the root apex, with retrograde preparation of 2–3 mm. (**D**) Retrograde filling with bioceramic iRoot BP plus. (**E**) Collection of CGF for gel preparation in the experimental group. (**F**) Preparation of the CGF membrane to cover the surface of the bone defect. (**G**) Suturing the incision. (**H**) Dissection of the lesion and pathological examination of the specimen, which revealed cyst wall-like tissue lined with squamous epithelium, with a hyperplasia of stromal fibrous connective tissue and extensive infiltration of chronic inflammatory cells.

The patient was positioned supine, with the side of surgical area supported by a shoulder pad to ensure comfort and adequate exposure of the operative field. Routine disinfection and draping were performed. The incision design for flap reflection was as follows: a local anesthesia was administered for all patients; the papilla base incision (PBI) was selected according to the location and extent of the jawbone lesion, with a trapezoidal incision adopted for the anterior region and an angular incision for the posterior region. For teeth receiving prosthetic restorations, a mucogingival incision was made 2–3 mm from the free gingiva to prevent gingival recession and preserve the aesthetic appearance of the prosthesis. A full-thickness flap was elevated along the affected tooth and adjacent teeth on both sides to fully expose the surgical field. Bone was removed to create a window and the periapical lesion was completely excised. Under the surgical microscope, 3 mm of the root apex was resected, followed by 2–3 mm retrograde root canal preparation. The root-end was filled with iRoot BP Plus bioceramic (Innovative Bio-Ceramix, Canada). The excised lesion underwent pathological examination. In the experimental group, the pre-prepared CGF was used. The red blood cell layer and plasma layer were aseptically removed, leaving the middle fibrin gel layer. A portion of the CGF gel was used to fill the bone defect, and a CGF membrane was placed over the surface of the bone defect, followed by wound closure and suturing. In the control group, the wound was directly closed and sutured after root-end filling (Figure 1A–H).

### Postoperative management and observation criteria

#### Postoperative pain and swelling assessment

The postoperative observation criteria included: (i) Postoperative complications, such as incision infection, wound dehiscence and bleeding, with the status of wound healing recorded; (ii) Postoperative pain assessment by the Numerical Rating Scale (NRS) [22] (0, no pain; 1–3, mild pain; 4–6, moderate pain; and 7–10, severe pain); (iii) Postoperative swelling, with the degree of swelling classified into four levels (A, no swelling; B, mild swelling, with swelling confined to the area from the alar of the nose to both corners of the mouth; C, moderate swelling, with swelling confined within the vertical line passing through the pupils; and D, severe swelling, with swelling extending beyond the aforementioned limits); (iv) Incision healing status, with the healing of the incision assessed on the 7th postoperative day during suture removal (Grade A, good incision healing, with no adverse reactions; Grade B, suboptimal healing, no suppuration of the incision; Grade C, occurrence of incision suppuration, requiring incision drainage or reopening of the wound).

#### Radiographic evaluation

CBCT images were taken immediately after the surgery and patients were instructed to attend regular follow-up visits at postoperative months 3, 6, 12 and 18. During each follow-up, the status of wound healing was examined and CBCT images were taken again. The data were imported in DICOM format into the Mimics Research version 20.0 software (Materialise HQ, Technologielaan, Leuven, Belgium) for 3D reconstruction. The bone defect volume was manually measured. All CBCT scans in this study were taken under the same conditions and interpreted by the same experienced radiologist.

### Data analysis

Based on the CBCT images, the following parameters were analyzed [23]:

- Absolute reduction in volume (AR): Initial volume—final volume.
- Relative reduction in volume (RR): (Initial volume—final volume) × 100/initial volume.
- Absolute speed of shrinkage (ASS): (Initial volume—final volume)/time.
- Relative speed of shrinkage (RSS): (Initial volume—final volume) × 100/initial volume × time.

The correlations between these four parameters and factors, such as patient age, gender and initial volume, were analyzed. As the observation lasted for 18 months for all cases, the results of volume reduction and shrinkage speed were compared between the two groups. A graph was plotted with time on the *x*-axis and relative speed of shrinkage on the *y*-axis to depict changes in bone defect volume.

### Three-dimensional reconstruction and morphological analysis

**Figure 2.**
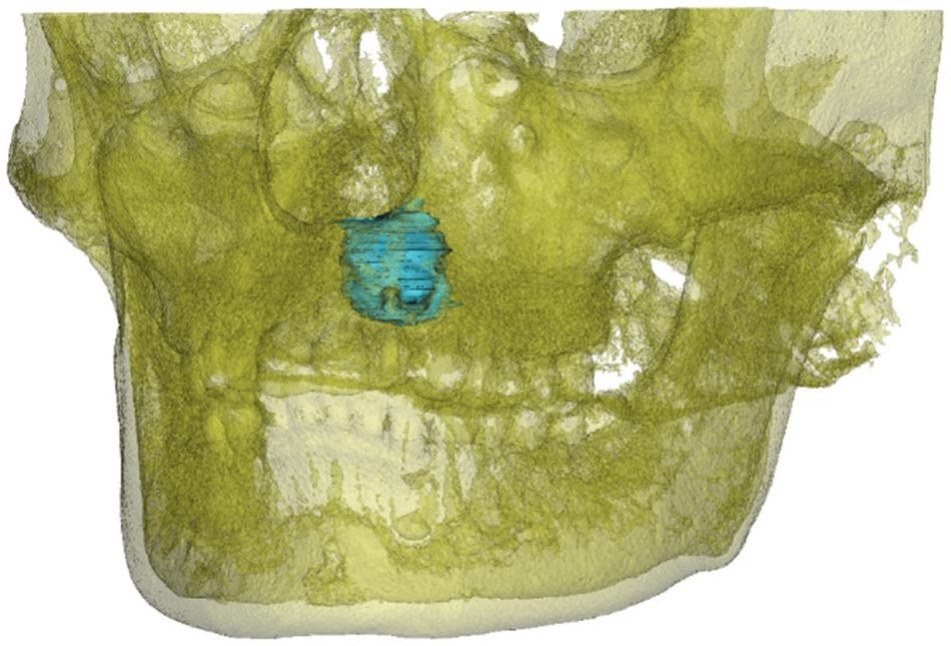
A three-dimensional model of root apical lesion within the jawbone reconstructed based on CBCT data.

- The CBCT data were imported into the Mimics 20.0 software. The window width and level were adjusted and an appropriate threshold (226–3000) was selected for segmentation. The results were saved as a “green” mask.
- A region-growing process was applied to the “green” mask to generate a “yellow” mask, during which most of the bone defect was segmented. A Boolean operation was adopted to obtain a “cyan” mask.
- The “cyan” mask was reviewed layer by layer and cavity filling was performed to obtain the final bone defect mask.
- The “Calculate part” tool in the software was employed to reconstruct the 3D model of the bone defect. The model was optimized by smoothing techniques.
- Clicking “Properties” displayed the bone defect volume of the 3D model (Figure 2). All measurements were performed jointly by two experienced microsurgical endodontists, with each measurement calculated twice and averaged.

### Statistical analysis

Statistical analysis was performed with SPSS 22.0. Continuous data with a normal distribution were expressed as mean ± standard deviation (*M* ± SD), and inter-group comparisons were evaluated by an independent samples *t*-test. For data without a normal distribution, the median (interquartile range) [M (IQR)] was used, and intergroup comparisons were analyzed by the Mann–Whitney *U* test. Categorical data were presented as *n* (%), with intergroup comparisons assessed by the chi-square test. The correlation between variables was examined by Spearman's correlation. A *P* value of less than 0.05 was considered statistically significant.

## Results

### Analysis of postoperative clinical outcomes

**Figure 3.**
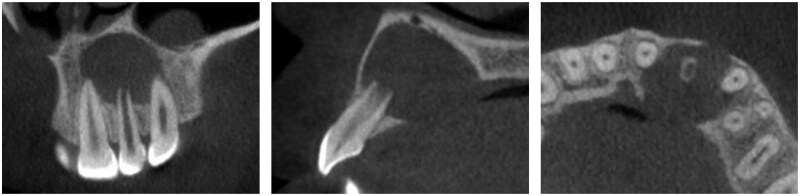
CBCT coronal, sagittal and axial views before surgery.

**Figure 4.**
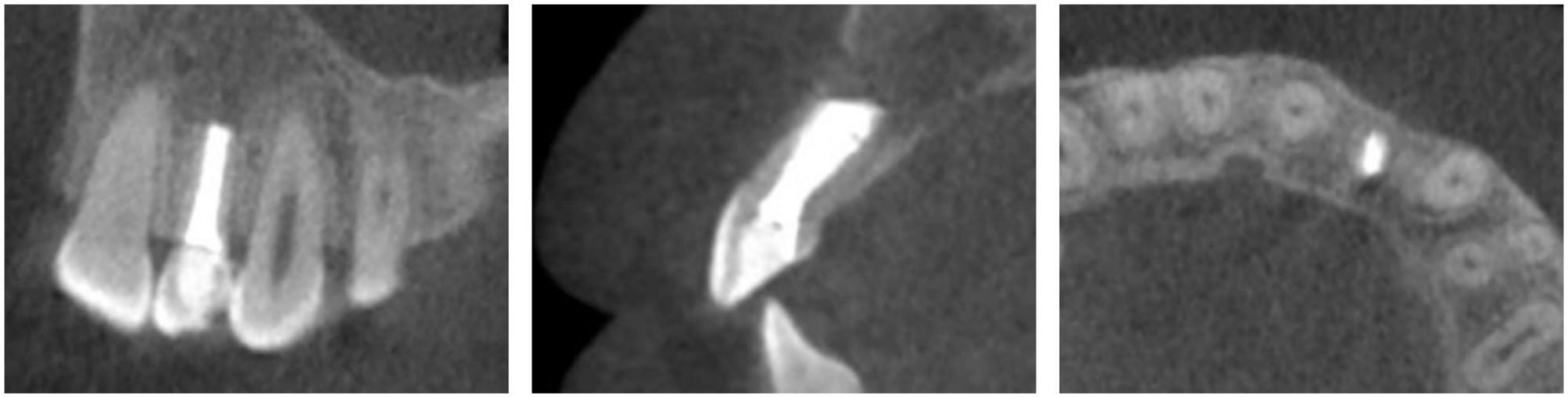
CBCT coronal, sagittal and axial views at postoperative month 6.

Postoperative pain, swelling and wound healing were comprehensively assessed for both the Experimental and Control groups. Compared with the Control group, the Experimental group reported a marked reduction in the incidence and duration of postoperative pain and the severity of swelling, with mild to moderate swelling commonly observed in the former but mild swelling predominated in the latter. Both groups reported good wound healing but significant differences in bone defect volume reduction and rate of bone regeneration. The CBCT images showed evident postoperative bone defect repair in both groups (Figures 3 and 4), with a faster new bone formation observed in the Experimental group than in the Control group.

#### Clinical group analysis

The differences in gender, age, immediate postoperative bone defect volume and maxillary/mandibular teeth (%) were explored between the Experimental and Control groups. The results are summarized in Table 1. The intergroup comparisons showed a significant difference in the immediate postoperative bone defect volume (*P* < 0.05) but no statistical differences between the two groups in the other aspects (*P* > 0.05).

**Table 1.** Baseline comparison between the two groups

Groups	*n*	Age （years）	Gender （%）		Immediate postoperative bone defect volume	Teeth （%）	
			Male	Female		Maxilla	Mandible
Control group	26	30.73 ± 12.84	4 (15.38)	22 (84.62)	98.1 (58.06, 258.12)	19 (73.08)	7 (26.92)
Experimental group	30	27.03 ± 9.85	11 (36.67)	19 (63.33)	204.85 (102.3, 450.96)	25 (83.33)	5 (16.67)
*X* ^2^/*t*		1.218	3.219		−2.316	0.870	
*P*		0.229	0.073		0.021	0.351	

#### Analysis of incidence and duration of postoperative pain

The incidence and duration of postoperative pain were analyzed in both the Experimental and Control groups. The results are detailed in Table 2. The intergroup comparisons reported significant differences in both pain incidence and duration, with lower postoperative pain incidence and shorter pain duration in the Experimental group than in the Control group.

**Table 2.** Comparison of postoperative pain incidence (P.P/d) and duration between the two groups

Groups	*n*	P.P/d （%）		P.P/d duration
		None	Present	
Control group	26	2 (7.69)	24 (92.31)	3 (2.5,4)
Experimental group	30	11 (36.67)	19 (63.33)	2 (1,2)
*X* ^2^/*z*		6.560		−3.915
*P*		0.010		<0.001

#### Analysis of incidence and duration of postoperative swelling

The incidence and duration of postoperative swelling were analyzed in both the Experimental and Control groups, with the results shown in Table 3. The intergroup comparisons indicated no significant difference in postoperative swelling incidence between the two groups. However, the difference in swelling duration was statistically significant. Compared with the control group, the experimental group reported a shorter duration of postoperative swelling, with swelling being primarily mild. In contrast, the control group experienced extended, mild to moderate swelling.

**Table 3.** Comparison of postoperative swelling incidence (P.S/d) and duration between the two groups

Groups	*n*	P.S/d （%）				P.S/d duration
		None	Mild	Moderate	Severe	
Control group	26	1 (3.85)	12 (46.15)	13 (50.00)	0 (0)	5 (4,7)
Experimental group	30	3 (10.00)	16 (53.33)	11 (36.67)	0 (0)	3 (1,3)
*z*		−1.142				−5.029
*P*		0.254				<0.001

#### Analysis of postoperative wound healing

Postoperative wound healing was analyzed between the Experimental and Control groups, with the results shown in Table 4. The intergroup comparisons revealed no statistical difference in wound healing at the time of suture removal. Both groups demonstrated good wound healing postoperatively.

**Table 4.** Comparison of wound healing at suture removal between the two groups

Groups	*n*	Suture removal healing （%）			*z*	*P*
		Grade A	Grade B	Grade C		
Control group	26	25 (96.15)	1 (3.85)	0 (0)	−1.074	0.283
Experimental group	30	30 (100)	0 (0)	0 (0)		

### Radiographic analysis

#### Analysis of radiographic bone volume ratio

After the intergroup comparison of the differences at various time points, the results, as shown in Table 5, indicated that the differences in bone volume at different time points were all statistically significant between the two groups.

**Table 5.** Comparison of differences between the two groups at various time points

Groups	*n*	Volume at 3 months	Volume at 6 months	Volume at 9 months	Volume at 12 months	Volume at 18 months
Control group	26	26.67 (16.12, 82)	44.42 (26.69, 138.36)	53.36 (32.32, 163.77)	59.83 (35.68, 194.68)	70.8 (37.24, 236.9)
Experimental group	30	109.26 (26.54, 207.05)	180.26 (73.96, 371.71)	192.43 (81.97, 420.22)	192.39 (95.91, 448.17)	195.61 (101.43, 448.33)
*z*		−2.464	−3.056	−3.351	−3.294	−3.187
*P*		0.014	0.002	0.001	0.001	0.001

**Figure 5.**
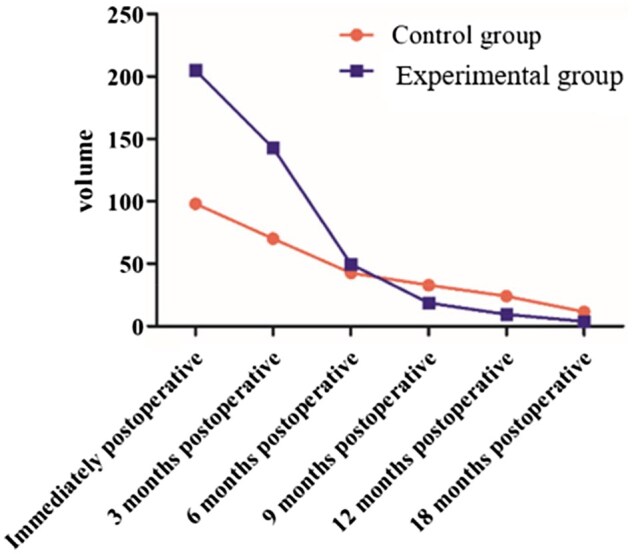
The curve of postoperative bone volume change.

As shown in Figure 5, a significant reduction in bone volume ratio was evident at postoperative month 3, indicating a decrease in bone defects and the formation of new bone. From postoperative month 6–18, the decrease in bone volume ratio was not significant. The new bone formation was the most active between postoperative month 3 and 6, which stabilized afterwards. By postoperative month 18, the bone defect reached its minimum size, with faster new bone formation and smaller bone defects in the Experimental group than in the Control group.

#### Analysis of volume reduction (VR) and volume reduction rate (VRR)

The results of the CBCT analysis of postoperative bone volume between the experimental and control groups are shown in Table 6. The intergroup comparisons revealed that the differences in volume reduction and reduction rate were statistically significant between the two groups, which indicates that CGFs substantially repair bone defects, with higher rates of new bone formation in the Experimental group than in the Control group.

**Table 6.** Comparison of VR and VRR between the two groups

Grouping	*n*	Absolute VR	Relative VR	Absolute VRR	Relative VRR
Control group	26	70.8 (37.24, 236.9)	86 (60.02, 96.73)	3.93 (2.07, 13.16)	4.78 (3.33, 5.37)
Experimental group	30	195.61 (101.43, 448.33)	97.61 (94, 99.6)	10.87 (5.64, 24.91)	5.42 (5.22, 5.53)
*z*		−3.187	−3.171	−3.187	−3.171
*P*		0.001	0.002	0.001	0.002

#### Correlation analysis

**Figure 6.**
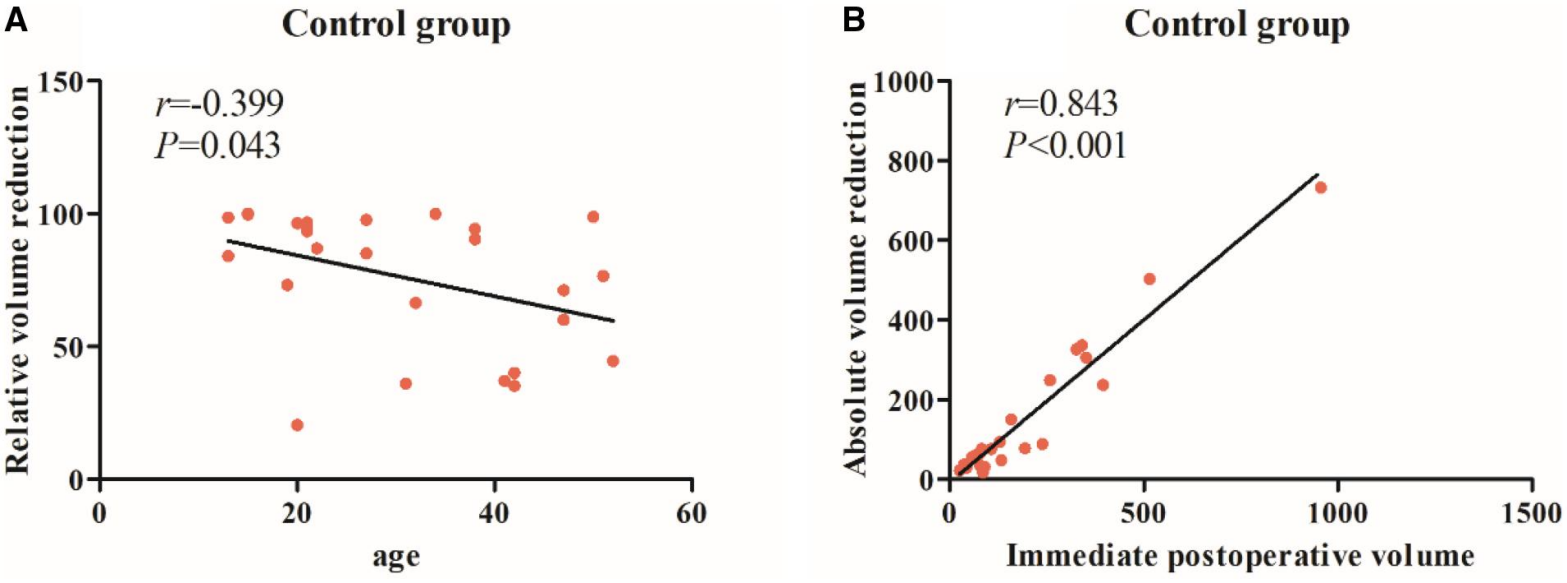
(**A**) The correlation analysis between age and relative volume reduction in the control group. (**B**) The correlation analysis between immediate postoperative volume and absolute volume reduction in the control group.

The correlation analysis of the Control group (Figure 6A) showed that age was negatively correlated with relative volume reduction and relative reduction rate, which indicates a faster postoperative bone volume reduction and quicker new bone formation in the younger patients. Additionally, immediate postoperative volume was positively correlated with absolute volume reduction and absolute reduction rate (Figure 6B), which suggests a significant reduction in bone defects and notable bone regeneration after apical surgery.

**Figure 7.**
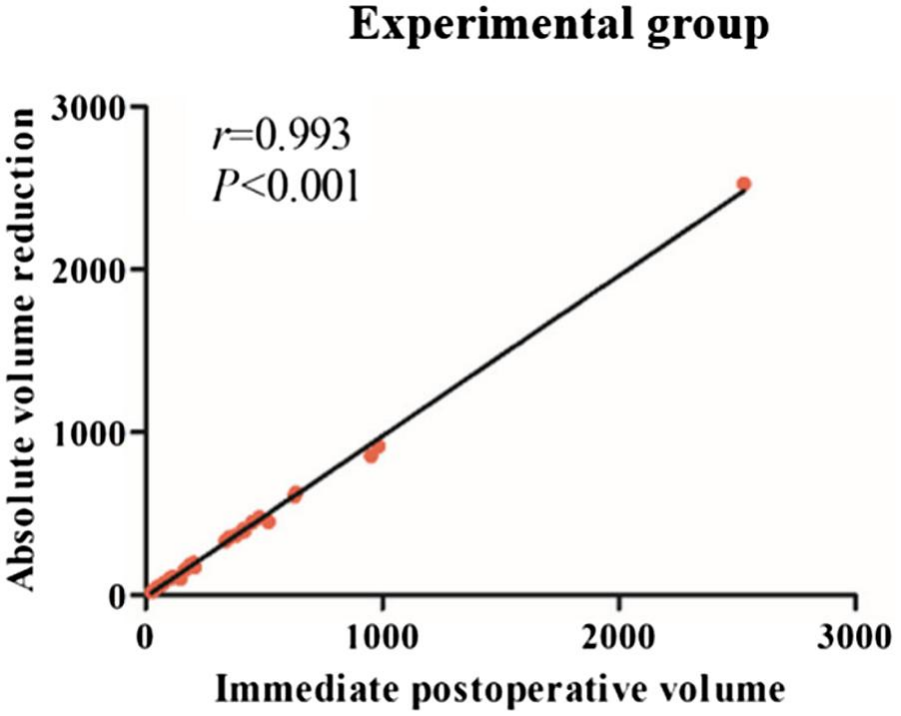
The correlation analysis between immediate postoperative volume and absolute volume reduction in the experimental group.

The correlation analysis of the Experimental group revealed no correlation of age with either relative volume reduction or relative reduction rate. However, immediate postoperative volume was positively correlated with absolute volume reduction and absolute reduction rate (Figure 7), which indicates significant improvement in bone defects after apical surgery.

## Discussion

Nonsurgical retreatment is the preferred option for periapical lesions. However, when infection is difficult to control, timely microsurgical endodontic surgery is required. Compared with traditional curettage techniques [24], microsurgical endodontic surgery embodies the principles of both minimally invasive and functional treatment. Previous studies have shown that the one-year success rate for microsurgical endodontic surgery can reach 90% or higher, with no statistical difference in outcomes between one postoperative year and two-ten postoperative years [25]. In this study, strict selection criteria for surgical indications, adherence to standardized operative protocols and an 18-month clinical follow-up were implemented to rigorously assess the efficacy of CGF in enhancing bone regeneration after microsurgical endodontic surgery.

In addressing periapical lesions, surgical trauma can alter the local microenvironment of the surrounding tissues and postoperative pain may often ensue from direct tissue damage. Postoperative pain, the duration of discomfort and swelling at the surgical site are important subjective indicators for evaluating the effectiveness of microsurgical endodontic surgery. In the present study, patients treated with autologous CGF reported significantly reduced postoperative pain and shorter pain duration, resulting in a higher acceptance and satisfaction with the surgery. These findings suggest that CGF not only offers biological advantages in tissue regeneration but also contributes positively to the reduction of postoperative pain.

In principle, tissue injury from surgical manipulation can activate local inflammatory responses that transmit pain signals to the central nervous system [26]. At the injury site, endothelial cells and macrophages may release various inflammatory mediators, such as histamine, bradykinin, prostaglandins and substance P [27], to act on nociceptors to lower the pain threshold and induce pain sensitization [28]. Among these mediators, interleukin-1β (IL-1β) can induce pain either by upregulating prostaglandin levels or directly stimulating nociceptors [29]. After surgical injury, activated monocytes may migrate to the damaged tissues and differentiate into macrophages with M1 or M2 phenotypes [30]. The former predominantly secretes pro-inflammatory cytokines to initiate immune responses, whereas the latter releases anti-inflammatory cytokines and growth factors to facilitate tissue repair and angiogenesis [31]. In addition, substance P released at the site of inflammation can induce macrophage polarization toward the M2 phenotype, thereby promoting healing [32].

Previous studies have shown that CGF contains abundant bioactive factors capable of promoting M2 macrophage polarization while reducing IL-1β expression, thus, effectively regulating the inflammatory response [33]. Consistently, the present study demonstrated that compared with those who did not receive CGF, patients treated intraoperatively with autologous CGF experienced a significantly lower rate and shorter duration of postoperative pain. The potential explanation for this observation may lie in that CGF alleviates postoperative discomfort by modulating the local immune microenvironment, potentially through the activation of the AKT signaling pathway. However, the precise molecular mechanisms underlying CGF-mediated immunomodulation warrant further investigation.

In terms of bone tissue repair, spontaneous healing alone often fails to meet the clinical demands for large bone defect regeneration. To date, the GBR technology, involving the use of autologous bone grafts or bone substitutes, has been widely employed in alveolar surgery, periodontology and implantology [34–37]. In this study, the application of CGF in GBR after the surgery for periapical lesions demonstrated its ability to promote bone regeneration and repair. These findings confirm that CGF is an effective biomaterial for tissue regeneration. CGF clots contain a significantly higher level of multiple growth factors, including vascular endothelial growth factor (VEGF), with VEGF concentration approximately 1.5 times higher in CGF than in PRF [38]. Studies have demonstrated that high concentrations of TGF-β, VEGF and PDGF can enhance osteogenesis and angiogenesis [39].

In terms of scaffold properties, compared with PRP and PRF, CGF forms a larger, denser fibrin network with superior cross-linking, providing a greater viscoelasticity and mechanical strength [40]. This robust autologous membrane can be independently used as a barrier membrane for soft tissue healing or as a natural scaffold for bone defect repair.

Biologically, CGF possesses a dense yet flexible fibrin network that facilitates the attachment, proliferation and differentiation of growth factors, osteoblasts and pre-osteoblasts [41]. With its osteoinductive and osteoconductive properties, CGF can promote the differentiation of undifferentiated mesenchymal cells into osteoblasts, facilitating the inward new bone formation. Additionally, CGF is rich in CD34+ cells and critical growth factors, including bone morphogenetic proteins (BMPs), VEGF, transforming growth factor-β (TGF-β), platelet-derived growth factor (PDGF), insulin-like growth factor (IGF) and fibroblast growth factor (FGF), all of which play pivotal roles in bone formation and remodeling. Specifically, BMP-2 can induce osteogenic differentiation of mesenchymal stem cells [42]; TGF-β and PDGF promote new bone formation while inhibiting osteoclastic bone resorption [43, 44]; and IGF regulates the differentiation and functional activities of osteoblasts and osteoclasts, contributing to bone remodeling and regeneration [45]. Moreover, the active protein factors within CGF can activate signaling pathways such as TGF-β/Smad, upregulating the expression of osteogenesis-related genes and proteins, thereby further enhancing bone regeneration [46].

In terms of angiogenesis, the high VEGF content in CGF promotes endothelial cell migration and proliferation, supporting the formation of new blood vessels and creating a favorable microenvironment for bone regeneration [47]. In addition, basic fibroblast growth factor (bFGF) accelerates endothelial cell repair and angiogenesis, thus, facilitating new bone formation and enhancing wound healing [48].

Collectively, the findings of this study demonstrate that compared with the counterparts, patients who received CGF-assisted GBR exhibited faster new bone formation after microsurgical endodontic surgery, with significant bone repair observed within three postoperative months. The subsequent decline in bone formation rates may be related to the degradation of CGF. With the release of osteoinductive factors and slower degradation sustained, the osteogenic potential of CGF for large bone defects may be even more pronounced.

Looking ahead, developing 3D personalized scaffolds for bone defect repair could provide a three-dimensional structure for cell adhesion and growth, resulting in better bone augmentation. Additionally, utilizing biomaterials to encapsulate growth factors could extend their release time, thereby enhancing osteogenesis, anti-inflammatory effects and angiogenesis. These approaches, which align with the physiological processes of bone regeneration and reconstruction, are expected to become key areas of research in bone tissue engineering.

Some limitations remain in this study: for one, CGF was used as the sole material for guided bone regeneration, which does not address the issue of rapid CGF degradation. Future studies could explore the combination of CGF with other bone tissue engineering materials, such as autologous bone or biomedical materials, to slow CGF degradation and achieve better bone defect repair outcomes. For another, randomized controlled trials could be considered to minimize bias introduced by non-randomized designs. For sample inclusion, it may be beneficial to standardize the use of single-tooth isolated bone defects as individual samples, which would reduce data discrepancies caused by comparing multiple-tooth defects with single-tooth defects.

## Conclusion

This study demonstrates that the use of CGF in guided bone regeneration after microsurgical treatment of periapical lesions leads to faster and stronger bone regeneration when compared with simple apical surgery with lesion removal. Furthermore, patients would experience less postoperative pain and swelling, making this a clinical technique worthy of broader promotion and application.

## References

[1] Ricucci D , Pascon EA, Ford TRP, Langeland K. Epithelium and bacteria in periapical lesions. Oral Surg Oral Med Oral Pathol Oral Radiol Endod 2006;101:239–49. 16448928 10.1016/j.tripleo.2005.03.038

[2] Ricucci D , Bergenholtz G. Histologic features of apical periodontitis in human biopsies. Endod Topics 2004;8:68–87.

[3] Mao WY , Lei J, Lim LZ, Tyndall DA, Fu K. Comparison of radiographical characteristics and diagnostic accuracy of intraosseous jaw lesions on panoramic radiographs and CBCT. Dentomaxillofacial Rad 2021;50:20200165. 10.1259/dmfr.20200165 PMC7860957 32941743

[4] Farmani AR , Nekoofar MH, Barough SE, Azami M, Rezaei N, Najafipour S, Ai J. Application of platelet rich fibrin in tissue engineering: focus on bone regeneration. Platelets 2021;32:183–88. 33577378 10.1080/09537104.2020.1869710

[5] Nanning LV , Zhou ZZ, Hou MZ, Hong LH, Li HY, Qian ZL, Gao XZ, Liu MM. Research progress of vascularization strategies of tissue-engineered bone. Front Bioeng Biotech 2024;11:1291969. 10.3389/fbioe.2023.1291969 PMC10834685 38312513

[6] Bruna L , Patricia S, Rui A, Mariana B, Ana S, CM, Luis MA, Ana CM. The application of mesenchymal stem cells on wound repair and regeneration. Appl Sci 2021;11:3000.

[7] Kawase T. Platelet-rich plasma and its derivatives as promising bioactive materials for regenerative medicine: basic principles and concepts underlying recent advances. Odontology 2015;103:126–35. 26040505 10.1007/s10266-015-0209-2

[8] Anitua E , Fernández-de-Retana S, Alkhraisat MH. Platelet rich plasma in oral and maxillofacial surgery from the perspective of composition. Platelets 2021;32:174–82. 33350883 10.1080/09537104.2020.1856361

[9] Murray T , Khetarpal S. Platelet-rich fibrin. Adv Cosmet Sur 2022;5:9–16.

[10] Lee HM , Shen EC, Shen JT, Fu E, Chiu HC, Hsia Y. Tensile strength, growth factor content and proliferation activities for two platelet concentrates of platelet-rich fibrin and concentrated growth factor. J Dent Sci 2020;15:141–46. 32595893 10.1016/j.jds.2020.03.011 PMC7305442

[11] Petrescu BN , Mirica IC, Miron R, Campian RS, Lucaciu O. Platelet rich fibrin as a gingival tissue regeneration enhancer. J Dent Sci 2021;16:536–39. 33384845 10.1016/j.jds.2020.08.014 PMC7770353

[12] Chen JG , Jiang HY. Clinical application of concentrated growth factor fibrin combined with bone repair materials in jaw defects. J Oral Maxil Surg 2020;78:1041. 10.1016/j.joms.2020.03.030 32360234

[13] Sun SF , Xu XD, Zhang ZX, Zhang Y, Wei WJ, Guo K, Jiang YN. A novel concentrated growth factor (CGF) and bio-oss based strategy for second molar protection after impacted mandibular third molar extraction: a randomized controlled clinical study. BMC Oral Health 2023;23:750. 37828455 10.1186/s12903-023-03411-2 PMC10571244

[14] Tabatabaei F , Aghamohammadi Z, Tayebi L. In vitro and in vivo effects of concentrated growth factor on cells and tissues. J Biomed Mater Res A 2020;108:1338–50. 32090458 10.1002/jbm.a.36906

[15] Yin XL , Shi H, Hze-Khoong EP, Hu YJ, Zhang CP. Effect of concentrated growth factor on distraction osteogenesis of dental implant distractors. J Oral Maxil Surg 2022;80:889–96. 10.1016/j.joms.2021.12.017 35240065

[16] Malcangi G , Patano A, Palmieri G, Pede CD, Latini G, Inchingolo AD, Hazballa D, Ruvo ED, Garofoli G, Inchingolo F, Dipalma G, Minetti E, Inchingolo AM. Maxillary sinus augmentation using autologous platelet concentrates (platelet-rich plasma, platelet-Rich fibrin, and concentrated growth factor) combined with bone graft: a systematic review. Cells 2023;12:1797. 37443831 10.3390/cells12131797 PMC10340512

[17] Zhang Z , Li X, Zhao J, Jia W, Wang Z. Effect of autogenous growth factors released from platelet concentrates on the osteogenic differentiation of periodontal ligament fibroblasts: a comparative study. PeerJ 2019;7:e7984. 31687282 10.7717/peerj.7984 PMC6825745

[18] Hong S , Li L, Cai W, Jiang B. The potential application of concentrated growth factor in regenerative endodontics. Int Endod J 2019;52:646–55. 30471228 10.1111/iej.13045

[19] Sureshbabu NM , Kathiravan S, Jayanth VK, Nandakumar M, Deepak S. Concentrated growth factors as an ingenious biomaterial in regeneration of bony defects after periapical surgery: a report of two cases. Case Rep Dent 2019;2019:1–6. 10.1155/2019/7046203 PMC6362495 30805222

[20] Yahata Y , Handa K, Ohkura N, Okamoto M, Ohshima J, Itoh S, Kawashima N, Tanaka T, Sato N, Noiri Y, Hayashi M, Okiji T, Saito M. Autologous concentrated growth factor mediated accelerated bone healing in root-end microsurgery: a multicenter randomized clinical trial. Regen Ther 2023;24:377–84. 37711762 10.1016/j.reth.2023.08.006 PMC10497983

[21] American Association of Endodontists (AAE). Guide to Clinical Endodontics, 6th edn. Chicago: American Association of Endodontists, 2016.

[22] Goulet J , Buta E, Carroll C. Statistical methods for the analysis of NRS pain data. J Pain 2015;16:7.

[23] Song IS , Park HS, Seo BM, Lee JH, Kim MJ. Effect of decompression on cystic lesions of the mandible: 3-dimensional volumetric analysis. Br J Oral Maxillofac Surg 2015;53:841–48. 26212420 10.1016/j.bjoms.2015.06.024

[24] Kim S , Kratchman S. Modern endodontic surgery concepts and practice: a review. J Endod 2006;32:601–23. 16793466 10.1016/j.joen.2005.12.010

[25] Setzer FC , Shah SB, Kohli MR, Karabucak B, Kim S. Outcome of endodontic surgery: a meta-analysis of the literature: part 1: comparison of traditional root-end surgery and endodontic microsurgery. J Endod 2010;36:1757–65. 20951283 10.1016/j.joen.2010.08.007

[26] Yam MF , Loh YC, Tan CS, Adam SK, Manan NA, Basir R. General pathways of pain sensation and the major neurotransmitters involved in pain regulation. Int J Mol Sci 2018;19:2164. 30042373 10.3390/ijms19082164 PMC6121522

[27] Malcangio M. Role of the immune system in neuropathic pain. Scand J Pain 2019;20:33–7. 31730538 10.1515/sjpain-2019-0138

[28] Zhang Y , Wang Y. TRPV 1: an important molecule involved in the peripheral sensitization during chronic pain and Central pain modulation. Sheng Li Xue Bao 2017;69:677–84. 29063115

[29] Aakanksha J , Irizarry-Caro RA, McDaniel MM, Chawla AS, Carroll KR, Overcast GR, Philip NH, Oberst A, Chervonsky AV, Katz JDP. T cells instruct myeloid cells to produce inflammasome independent IL-1 13 and cause autoimmunity. Nat Lmmunol 2020;21:65–74. 10.1038/s41590-019-0559-y PMC6927526 31848486

[30] Ginhoux F , Jung S. Monocytes and macrophages: developmental pathways and tissue homeostasis. Nat Rev Immunol 2014;14:392–404. 24854589 10.1038/nri3671

[31] Rőszer T. Understanding the mysterious M2 macrophage through activation markers and effector mechanisms. Mediat Inflamm 2015;2015:1–16. 10.1155/2015/816460 PMC4452191 26089604

[32] Jetten N , Verbruggen S, Gijbels MJ, Post MJ, De Winther MPJ, Donners MMPC. Anti-inflammatory M2, but not pro-inflammatory M1 macrophages promote angiogenesis in vivo. Angiogenesis 2014;17:109–18. 24013945 10.1007/s10456-013-9381-6

[33] Haiyun L , Wenjing L, Yachuan Z, Jiang X, Liu YY, Yang Q, Shao LQ. Concentrated growth factor regulates the macrophage-mediated immune response. Regen Biomater 2021;8:049. 10.1093/rb/rbab049 PMC8421811 34513006

[34] Inoh C , Jaeha B, Byungock K, Sangjoun Y, Keonil Y, Wonpyo L. Evaluation of the effect of three‐dimensional, preformed titanium membrane on peri‐implant extrabony defects for simultaneous GBR with implantation. Clin Oral Implants Res 2019;30:416.

[35] Takallu S , Mirzaei E, Zakeri BA, Jafarbeigloo HRG, Khorshidi H. Addressing antimicrobial properties in guided tissue/bone regeneration membrane: enhancing effectiveness in periodontitis treatment. ACS Infect Dis 2024;10:779–807. 38300991 10.1021/acsinfecdis.3c00568

[36] Yao MY , Hu JJ, Jiang L, Guo R, Wang XM. Efficacy of concentrated growth factor combined with grafting materials vs. grafting materials alone for the treatment of periodontal intrabony defects: a systematic review and meta-analysis. Ann Transl Med 2023;11:184. 36923076 10.21037/atm-23-891 PMC10009557

[37] Eitan M , Haya DA, Oren P, Maayan S, Loredana C, Luca M. Use of PRP, PRF and CGF in periodontal regeneration and facial rejuvenation—a narrative review. Biology (Basel) 2021;10:317. 33920204 10.3390/biology10040317 PMC8070566

[38] Park HC , Kim SG, Oh JS, You JS, Kim JS, Lim SC, Jeong MA, Kim JS, Jin S, Jung C, Kwon YS, Ji H. Early bone formation at a femur defect using CGF and PRF grafts in adult dogs. Implant Dent 2016;25:387–93. 27123893 10.1097/ID.0000000000000423

[39] Chen X , Wang J, Yu L, Zhou J, Zheng DN, Zhang B. Effect of concentrated growth factor (CGF) on the promotion of osteogenesis in bone marrow stromal cells (BMSC) in vivo. Sci Rep 2018;8:5876. 29651154 10.1038/s41598-018-24364-5 PMC5897572

[40] Ren SC , Wang HC, Ma SJ, Zhou J, Zhai JJ, Zhu YM, Chen S, Chen SY, Jia KW, Xu WZ, Zhou YM. New strategy of personalized tissue regeneration: when autologous platelet concentrates encounter biomaterials. Front Bioeng Biotechnol 2023;11:1297357. 38076421 10.3389/fbioe.2023.1297357 PMC10698744

[41] Zhang LL , Ai HJ. Concentrated growth factor promotes proliferation, osteogenic differentiation, and angiogenic potential of rabbit periosteum-derived cells in vitro. J Orthop Surg Res 2019;14:146. 31118077 10.1186/s13018-019-1164-3 PMC6532180

[42] Onji K , Kabir MA, Zhu B, Yokozeki K, Saito T, Akazawa T, Murata M. Human fresh fibrin membrane with bone morphogenetic protein-2 (BMP-2) induces bone formation in the subcutaneous tissues of nude mice. Materials 2020;14:150. 33396335 10.3390/ma14010150 PMC7796051

[43] Salkin H , Acar MB, Korkmaz S, Gunaydin Z, Gonen ZB, Basaran KE, Ozcan S. Transforming growth factor β1-enriched secretome up-regulate osteogenic differentiation of dental pulp stem cells, and a potential therapeutic for gingival wound healing: a comparative proteomics study. J Dent 2022;124:104224. 35843478 10.1016/j.jdent.2022.104224

[44] Wang F , Ye Y, Zhang ZJ, Teng WSY, Sun HX, Chai XP, Zhou XZ, Chen JY, Mou HC, Eloy YW, Jin XQ, Chen L, Shao ZX, Wu Y, Shen Y, Liu A, Lin P, Wang JW, Yu XH, Ye ZM. PDGFR in PDGF-BB/PDGFR signaling pathway does orchestrates osteogenesis in a temporal manner. Research 2023;6:86. 10.34133/research.0086 PMC10202377 37223474

[45] Zhou WL , Li LL, Qiu XR, An Q, Li MH. Effects of combining insulin-like growth factor 1 and platelet-derived growth factor on osteogenesis around dental implants. Chin J Dent Res 2017;20:105–09. 28573264 10.3290/j.cjdr.a38275

[46] Wang LQ , Ruan MJ, Bu QQ, Zhao CZ. Signaling pathways driving MSC osteogenesis: mechanisms, regulation, and translational applications. Int J Mol Sci 2025;26:1311. 39941080 10.3390/ijms26031311 PMC11818554

[47] Lopes H , Gomes MP, Lima J, Quiles G, Totoli G, Freitas G, Beloti M, Rosa A. Osteogenic effect of the association of VEGF-A and BMP-9 on mesenchymal stem cells. Bone Rep 2020;13:100437.

[48] Kang WY , Liang QY, Du LQ, Shang LL, Wang T, Ge SH. Sequential application of bFGF and BMP‐2 facilitates osteogenic differentiation of human periodontal ligament stem cells. J Periodontal Res 2019;54:424–34. 30851068 10.1111/jre.12644

